# Integrated Analysis Reveals Functional Ingredients Biosynthesis and Regulatory Mechanisms in *Elaeagnus mollis* Diels Leaf

**DOI:** 10.1002/fsn3.71679

**Published:** 2026-03-25

**Authors:** Hu Xiaoyan, Su Yanping, Song Jia, Wu Jing, Qiu Qiandong, Yu Wendong, Wu Zhenzhen, Huang Yuyin, Du Shuhui

**Affiliations:** ^1^ College of Food Science and Engineering Shanxi Agricultural University Jinzhong Shanxi China; ^2^ College of Life Science Langfang Normal University Langfang Hebei China; ^3^ Powerchina Northwest Engineering Corporation Limited Shaanxi China; ^4^ Shanxi Academy of Forestry and Grassland Taiyuan Shanxi China; ^5^ College of Agriculture and Biology Liaocheng University Liaocheng Shandong China; ^6^ College of Horticulture and Landscape Architecture Yangzhou University Yangzhou Jiangsu China; ^7^ Shandong Huinongtianxia Science and Technology Information Consulting Co., Ltd Taian Shandong China; ^8^ College of Forestry, Shanxi Key Laboratory of Efficient Cultivation of Forest Resources Shanxi Agricultural University Jinzhong Shanxi China

**Keywords:** antioxidant capacity, co‐expression network, comprehensive multi‐omics analysis, *Elaeagnus mollis* leaf, functional ingredients

## Abstract

*Elaeagnus mollis* Diels is a newly developed woody oil crop, and the biosynthesis and regulatory mechanism underlying functional and nutritional ingredients in leaf have long been neglected. In the present study, we integrated quantification and multi‐omics analysis to unravel the biosynthesis pathway and regulatory mechanism of flavonoids, phenols, and terpenoids, which all held substantial nutritional significance in *E. mollis* leaf (EML). Quantification results indicated that the contents of three ingredients increased with EML development and showed fluctuations before senescence, with the highest value as 27.45 mg/g for flavonoids and 17.486 mg/g for phenols. The accumulation of these functional ingredients contributed primarily to the in vitro antioxidant capacity, which directly reflected the potential nutritional utilization of EML. Comprehensive analysis of multi‐omics data identified 14, 19, and 37 DEGs, as well as 32, 21, and 52 DAMs involved in the biosynthesis pathway of flavonoids, phenols, and terpenoids, respectively. Co‐expression network construction revealed that transcription factors from *EmNACs*, *EmMYBs*, and *EmDREBs* may regulate the biosynthesis of flavonoids, phenols, and terpenoids. The specific positive regulatory roles of *EmNAC014* and *EmMYB15* in flavonoids and phenols biosynthesis were verified with in vivo transient overexpression and VIGS analysis. Furthermore, some potential coregulation factors simultaneously participating in various biosynthesis pathways were identified. This study not only expands the acknowledgement of functional ingredients biosynthesis and accumulation pattern, but also provides vital biomarkers that can be targeted for regulating the contents to enhance the nutritional value and utilization of *E. mollis* leaf.

## Graphical abstract

The content of functional ingredient fluctuated in *E. mollis* leaf. The functional ingredients biosynthesis pathways were revealed. *EmNAC014* and *EmMYB15* played key roles in functional ingredients biosynthesis.

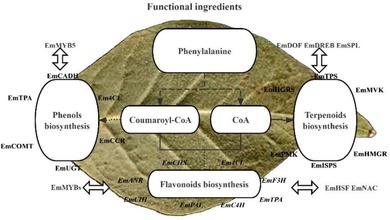

*Elaeagnus mollis* Diels, a deciduous species in the family Elaeagnaceae, is classified as endangered by both IUCN and IPLANT, and naturally occurs only on the Loess Plateau in China (Du et al. 2025). The National Health and Family Planning Commission of China approved *E. mollis* kernel oil as new resource food, and *E. mollis* has been promoted as a novel woody oil crop since then. Except for the high content of unsaturated fatty acid (Kan et al. 2017; Li, Xiaoyan, et al. 2025; Liang et al. 2015; Wang et al. 2019; Wu et al. 2020), various functional ingredients, such as vitamin E and flavonoids, have been reported to be rich in *E. mollis* kernel (Du et al. 2024; Feng et al. 2025; Li et al. 2024). These ingredients are widely recognized for their health‐promoting effects, including modulation of blood lipids and pressure, and protection of cardiovascular health (Dias et al. 2021; Tao et al. 2024). This beneficial composition makes *E. mollis* a promising candidate for functional food and nutrition applications. For instance, *E. mollis* oil capsule obtained the health food approval certificate from the State Food and Drug Administration of China (2003–0403) and was launched in 2003 with the aim to regulate blood lipid.

The exploration of functional ingredients content dynamics and underlying biosynthesis regulatory mechanism has been widely conducted in *E. mollis* kernel; the biosynthesis pathway, functional genes, and significant regulators have been identified and verified (Du et al. 2024, 2025; Feng et al. 2025; Li et al. 2024). With the development of a full, healthy, and encompassing approach to food, the exploration and utilization of whole plants have been popular. For instance, the biosynthesis and accumulation pattern of lipids, amino acids, and flavonoids in seeds, leaves, and floral of *Camellia hainanica* were addressed, which provided valuable support for the higher nutritional value and expanded the development and utilization of camellia oil (Ding et al. 2025). Total flavonoids and phenols contents in American ginseng fruits were significantly higher than those in roots, which offered valuable insights for enhancing the development and utilization of American ginseng fruits as functional foods (Zhang, Hou, et al. 2025). As in *E. mollis* , beyond the related researches concerning functional ingredients in kernel, these compounds in leaves have long been neglected. Several researches have revealed that *E. mollis* leaves (EML) are a rich source of various biologically active and nutritional compounds including flavonoids, phenols, and terpenoids, which all harbor bioactive characteristics such as antioxidant and anti‐disease (Li et al. 2015; Liu et al. 2012). The extracts from EML illustrated the inhibitory activity against cholinesterase and HepG2 cancer cells, and could be used as an effective source of functional components to provide benefits to physical health care and the food industry (Li et al. 2020). But until now, little is available concerning the biosynthesis and regulatory mechanism of these compounds, which limits the application of EML in the food‐ and nutrition‐related industry.

In the present study, we employed quantification and multi‐omics methods to determine the flavonoids, phenols, and terpenoids contents in EML at various development stages to elucidate the accumulation pattern, biosynthesis pathway, and regulatory mechanisms. The findings of the present study not only provide novel insights into the regulatory mechanisms underlying the biosynthesis of key functional components but also serve as a theoretical basis for the fully development and utilization of *E. mollis* .

## 1 Materials and Methods

### 1.1 Plant Material and Sample Collection

**FIGURE 1:**
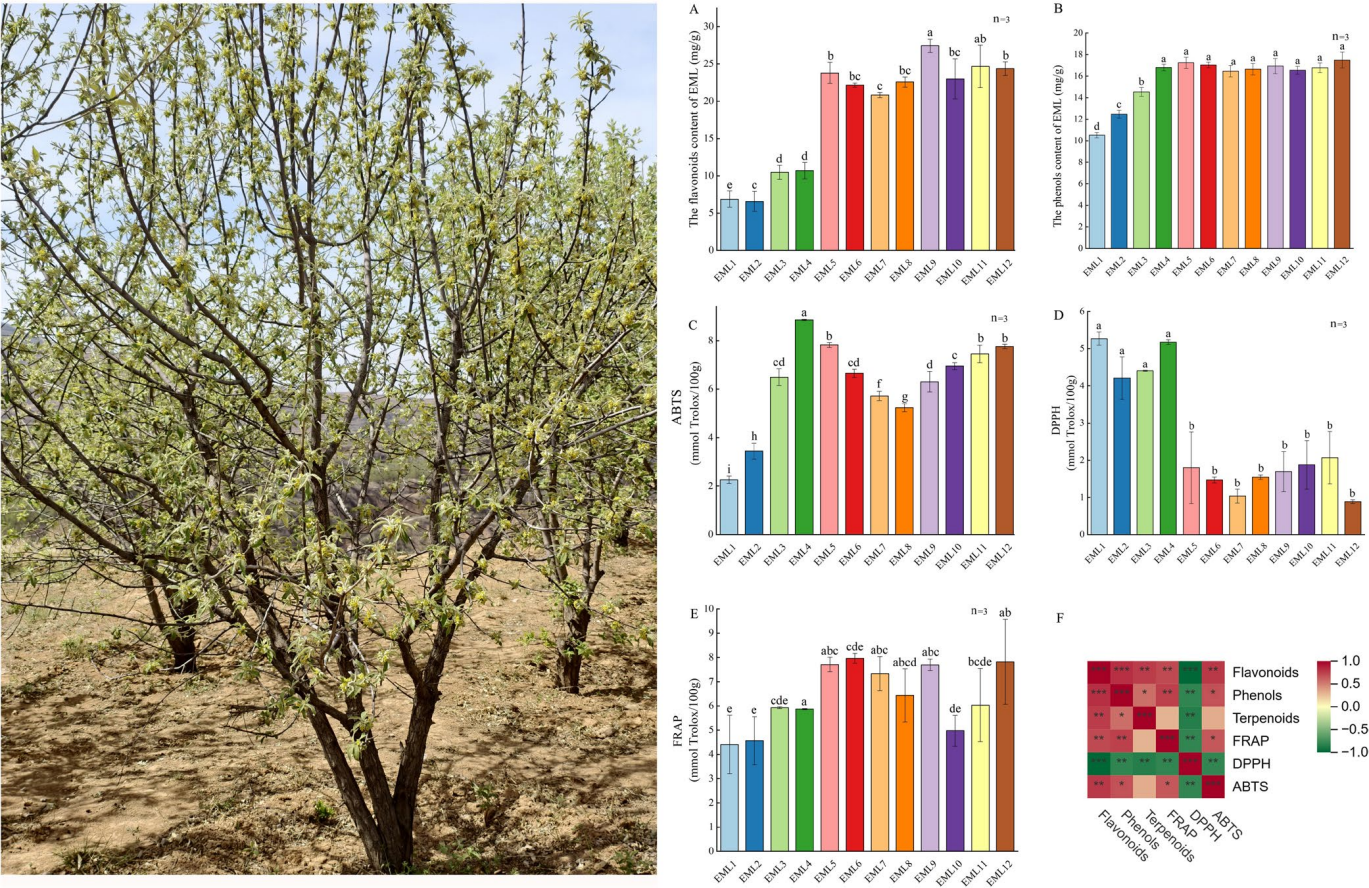
The functional ingredients contents, antioxidant capacities and the correlation analysis between these measured parameters of EML. (A) The content of flavonoids; (B) The content of phenols; (C) the ABTS capacity; (D) the DPPH capacity; (E) the FRAP capacity; (F) correlation between the contents of functional ingredients and antioxidant capacities, lowercase indicated significant difference (*p < 0.05, **p < 0.01; ***p < 0.001).

Leaf samples were collected from three healthy and well‐developed 20‐year‐old *E. mollis* trees in Xiangning, Shanxi, China from 15 April to 30 September 2024 for per 15 days and hereafter referred as EML1‐EML12 (Figure 1). These trees were planted in the local garden of *E. mollis* ‐related enterprise using artificially cultivated seedlings. Samples covering top‐down leaves of each individual were equally mixed and divided into two portions. One portion was immediately frozen in liquid nitrogen and kept at −80°C, while the left was taken back to the laboratory for compounds determination.

### 1.2 Functional Ingredient Determination and Antioxidant Capacity

The content dynamics of flavonoids, phenols and the overall bio‐activity of EML were determined to set a solid foundation for future utilization and exploration in food and nutrition industry. Total flavonoids content (TFC) was quantified using the assay kit BC1330 (Beijing Solarbio Science & Technology Co. Ltd) according to the manufacturer's instruction. Briefly, 0.1 g fresh EML was dried at 60°C to constant weight, ground, and sieved at a 40‐mesh screen. The powder was extracted with 1 mL 2% aluminum chloride solution using ultrasound extraction in a water bath at 300 Hz for 30 min followed by centrifugation at 12,000 rpm for 10 min at 25°C. The absorbance of the mixture was measured at 470 nm and TFC was calculated as rutin equivalents (mg/g dry weight of EML). The determination was done three times for every sample. Total phenols content (TPC) was determined using the assay kit BC1330 (Beijing Solarbio Science & Technology Co. Ltd) according to the guideline. The 0.1 g EML powder was extracted with 2.5 mL 50% Folin–Ciocalteu reagent (v/v) as the process of TFC determination. The absorbance of the mixture was measured at 760 nm and TPC was illustrated as gallate equivalents (mg/g dry weight of EML). Triplicates were conducted for every determination. The total in vitro antioxidant capacity of EML was determined using 2,2‐diphenyl‐1‐picryl‐hydrazyl‐hydrate (DPPH), ferric reducing antioxidant power (FRAP), 2,2‐azinobis (3‐ethylbenzothiazoline‐6‐sulfonic acid) diammonium salt (ABTS) assay kits from Suzhou Grace following the protocol. Totally, 1 g air‐dried EML was extracted with 15 mL acidified methanol (0.1% HCl v/v) and the extract was used in the following antioxidant analysis based on the instruction. Results were expressed as Trolox equivalent (TE) antioxidant capacity. All the antioxidant analyses were conducted with three replicates.

### 1.3 Metabolome Analysis

For metabolites extraction, 100 mg EML were grounded with liquid nitrogen and re‐suspended in ice‐cold 80% methanol that contained a cocktail of internal standards, followed by vigorous vortex. After 5 min incubation on ice, the mixture was centrifuged at 15,000 *g*, 4°C for 20 min. An aliquot of the supernatant was brought to 53% methanol (v/v) with LC–MS grade water, and re‐centrifuged under the same conditions. The resulting supernatant was injected directly into the LC–MS/MS system for analysis, which were performed with an ExionLC AD system (SCIEX) coupled with a QTRAP 6500^+^ mass spectrometer (SCIEX) in Novogene Co. Ltd. (Beijing, China). Sample was injected into an Xselect HSS T3 (2.1 × 150 mm, 2.5 μm) using a 20 min linear gradient at a flow rate of 0.4 mL/min for the polarity mode. The eluents consisted of A (0.1% formic acid‐water) and B (0.1% formic acid‐acetonitrile). The solvent gradient was set as follows: 2% B, 2 min; 2%–100% B, 11.0 min; 100% B, 13.0 min; 100%–2% B, 13.1 min; 2% B, 15 min. Afterwards, QTRAP 6500^+^ mass spectrometer was operated in positive polarity mode with curtain gas of 35 psi, collision gas of medium, ions pray voltage of 5500 V, temperature of 550°C, ion source gas of 1:60, ion source gas of 2:60, and in negative polarity mode with curtain gas of 35 psi, collision gas of medium, ion spray voltage of −4500 V, temperature of 550°C, ion source gas of 1:60 and ion source gas of 2:60. The detection of the experimental samples using MRM (Multiple Reaction Monitoring) were in line with Novogene in‐house database. The Q3 were used to the metabolite quantification and the Q1, Q3, RT (retention time), DP (declustering potential) and CE (collision energy) were used to the metabolite identification. These metabolites were annotated using the KEGG, HMDB and Lipidmaps databases. Principal components analysis (PCA) and partial least squares discriminant analysis (PLS‐DA) were performed at metaX (Luo et al. 2015) to determine the reliability of the metabolome data. Moreover, univariate analysis (*t*‐test) was applied to calculate the statistical significance (*p*‐value). The metabolites with VIP > 1 and *p* < 0.05 and fold change (FC) ≥ 2 or ≤ 0.5 were considered to be differential accumulated metabolites (DAMs). These DAMs underwent further analysis for KEGG pathway enrichment with a significance threshold of *p* < 0.05. Three replicates were used in metabolome analysis.

### 1.4 Transcriptome Sequencing and Analysis

EML samples of 100 mg were snap‐frozen in liquid nitrogen and homogenized, after which total RNA was isolated with TRIzol reagent (Thermo Fisher). Stranded libraries were prepared and paired‐end reads (150 bp) were generated on an Illumina NovaSeq 6000. Adapters and low‐quality bases were removed with Trimmomatic (Bolger et al. 2014), cleaned reads were then mapped to the self‐assembled *E. mollis* genome using HISAT2 v2.2.1 (Kim et al. 2019) to conduct annotation. Transcripts were assembled *de novo* with StringTie, merged across samples, and quantified as FPKM. Genes with |log_2_FC| ≥ 1 and FDR < 0.05 were called differentially expressed (DEGs). KEGG enrichment analysis was carried with KOBAS‐i (Bu et al. 2021). Transcript abundance was verified by qRT‐PCR validation on a LightCycler 480 instrument (Roche) with a SYBR‐based master mix (Vazyme, Nanjing). Fold changes were derived with the 2^−ΔΔCt^ algorithm. Primers used were supplied in Table S1. Furthermore, multivariate patterns were explored by PCA and Pearson's correlations to testify the repeatability and reliability of the transcriptome data. Three replicates were used in transcriptome and qRT‐PCR analysis.

### 1.5 Verification With Transient Overexpression and Virus‐Inducted Gene Silencing in EML

Total RNA was extracted from 100 mg liquid frozen EML5 and inversed transcription into cDNA using the FastKing gDNA Dispelling RT SuperMix (KR118, Tiangen, Beijing, China). Then, full‐length CDS of *EmNAC014* and *EmMYB15* was amplified and inserted into pBI121 and pTRV2 (with pTRV1 as the helper plasmid) to produce overexpression and silencing constructs, respectively. Transient overexpression and Verification With Transient Overexpression and Virus‐Inducted Gene Silencing (VIGS) constructs were introduced into five fully developed leaves of 2‐year‐old *E. mollis* seedlings via *Agrobacterium*‐mediated vacuum infiltration, respectively. Transformed leaves were incubated in the dark for 48 h and transferred to a growth chamber for 24 h. Flavonoids and phenols contents were quantified in the tissue surrounding all the injection sites of three independent leaves using the above‐mentioned methods. Primers used for amplification and plasmid construction are listed in Table S1.

### 1.6 Statistically Analysis

To uncover TFs involved in flavonoids, phenols, and terpenoids biosynthesis in EML, we employed Pearson's correlation among all the identified differentially expressed TFs, corresponding DEGs, and DAMs using the Novogene Cloud. Results with coefficients |*R*| ≥ 0.8 and *p* ≤ 0.05 were selected and combined to construct a co‐expression network using Cytoscape 3.10.0 (Killcoyne et al. 2009). We employed R and IBM SPSS software for the statistical analyses of all data. One‐way analysis of variance (ANOVA) and Duncan's tests were used to assess the significant differences. Origin was utilized to produce all the graphics.

## 2 Results

### 2.1 Functional Ingredients Contents Dynamics in EML

Flavonoids and phenols contents illustrated various accumulation patterns throughout EML development. TFC increased along with EML development to the highest of 27.45 mg/g at EML9, and decreased a little when mature (Figure 1A). TPC initially increased to EML4 and maintained a relatively stable level without significant fluctuation hereafter until it reached the highest value of 17.486 mg/g at EML12 (Figure 1B). However, the absolute content of terpenoids cannot be determined, so we used the normalized relative content from metabolome data in subsequent analysis. Results showed that the relative content of terpenoids was the highest in EML9, as the lowest in EML3 (Figure S1).

### 2.2 Antioxidant Capacity of EML

The in vitro ABTS, DPPH, and FRAP scavenging capacities of EML extracts showed a significant discrepancy with each other (Figure 1C–E). Pearson's correlation analysis with functional ingredients contents and antioxidant scavenging capacities revealed that flavonoids and phenols contents showed significant association with ABTS and FRAP capacities, and relative terpenoids content showed no correlation with ABTS and FRAP capacities. Noticeably, a significantly negative correlation between DPPH and all functional ingredients contents was revealed (Figure 1F).

### 2.3 Metabolites in EML

Pearson's correlation analysis and PCA results indicated that the metabolome data were reliable for the following analysis (Figure S2A,B). We identified a total of 1332 metabolites composed of 12 major groups in EML. Among them, phenylpropanoids and polyketides accounted for the highest proportion (281 metabolites, 21.10%) followed by organic acids and derivatives (270, 20.27%) and lipids and lipid‐like molecules (192, 14.41%) (Figure S2C). A number of 468, 441, and 406 DAMs were identified across the three comparison groups (EML3/EML5, EML5/EML9, and EML9/EML12), within which 199, 202, and 337 were up‐accumulated while 269, 239, and 69 were down‐accumulated, respectively. There were 31 common DAMs shared among the three comparisons, which included nine flavonoids, five phenols and four terpenoids, etc. These metabolites had the potential to influence the overall quality, nutritional composition, and other pertinent characteristics of EML, thereby can serve as biomarkers across EML development. KEGG enrichment analysis illustrated that the DAMs were related to multiple metabolic pathways (Figure S3). Specifically, flavonoid biosynthesis, cutin, suberine and wax biosynthesis were enriched in early EML development stages, while biosynthesis of secondary metabolites, monoterpenoid biosynthesis in late stages. These enriched pathways contributed primarily to the biosynthesis of these three functional ingredients in EML.

### 2.4 Transcriptome Analysis

The results of PCA, correlation and qRT‐PCR analysis indicated the credibility and repeatability of the transcriptome data for EML (Figure S4). We identified 493, 911, and 440 DEGs in the three comparison groups, within which 179, 710, and 18 DEGs were significantly up‐regulated, while 314, 201, and 422 down‐regulated, respectively. Noticeably, the number of DEGs in EML5/EML9 was much higher than that in the other two comparisons, which suggested that the development of EML may be drastically changed before senescence. The identified DEGs were enriched in various KEGG biosynthetic and metabolic pathways, such as flavonoid biosynthesis, biosynthesis of secondary metabolites (Figure S5). Furthermore, a comprehensive analysis of TFs using PlantTFDB and PlnTFDB resulted in the identification of 1720 TFs consisting of 144 DEGs (tfDEGs) belonging to 40 families, including 19 MYBs, 10 AP2/ERFs, etc. (Table S2). TFs from various families exhibited distinct expression patterns during EML development, which resulted from the various regulatory roles of specific TF members.

### 2.5 Functional Ingredients Biosynthesis in EML

**FIGURE 2:**
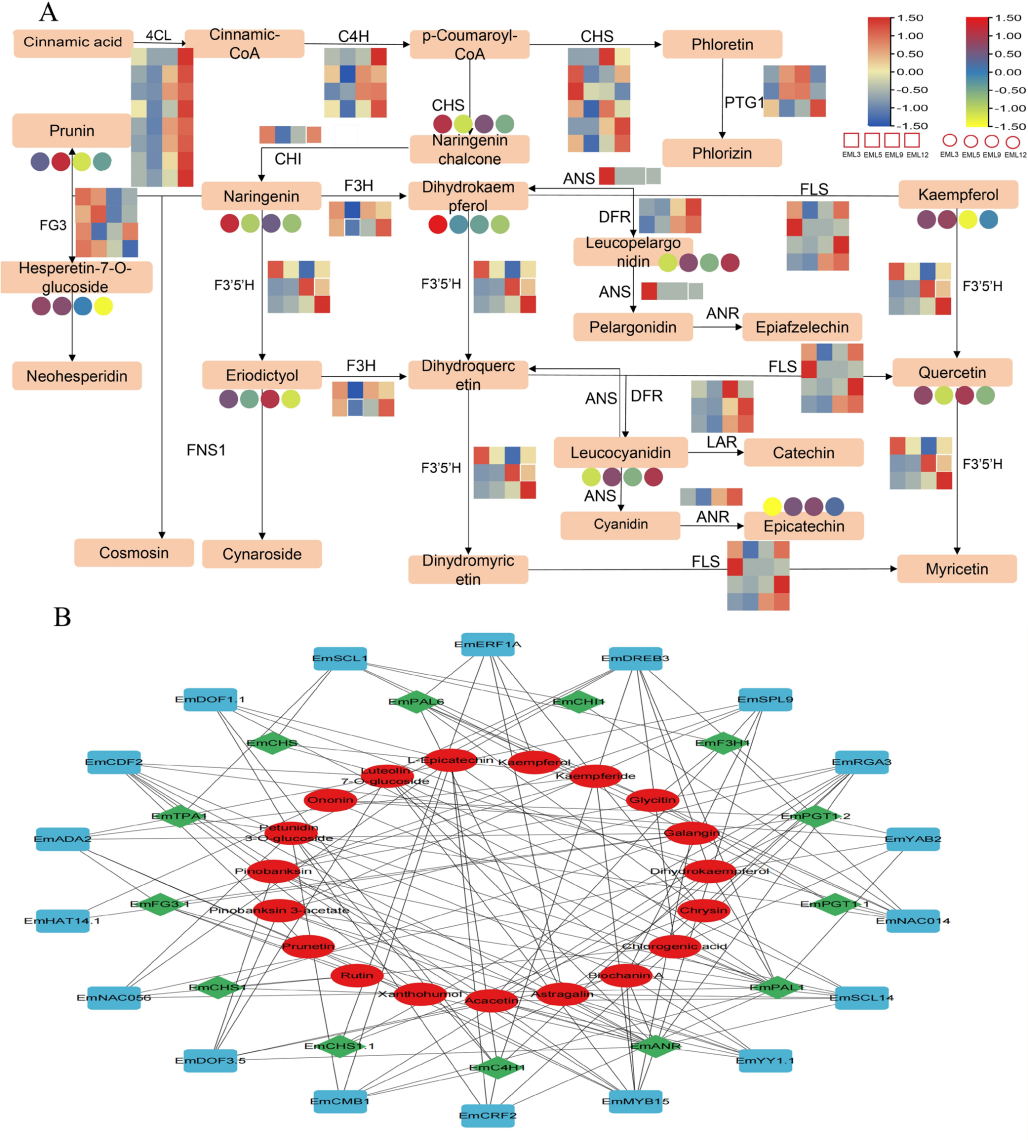
The biosynthesis pathway (A) and co‐expression network (B) of flavonoids in EML. Blue square indicated differentially expressed transcription factors, greed rhombus indicated DEGs and red circles indicated DAMs.

**FIGURE 3:**
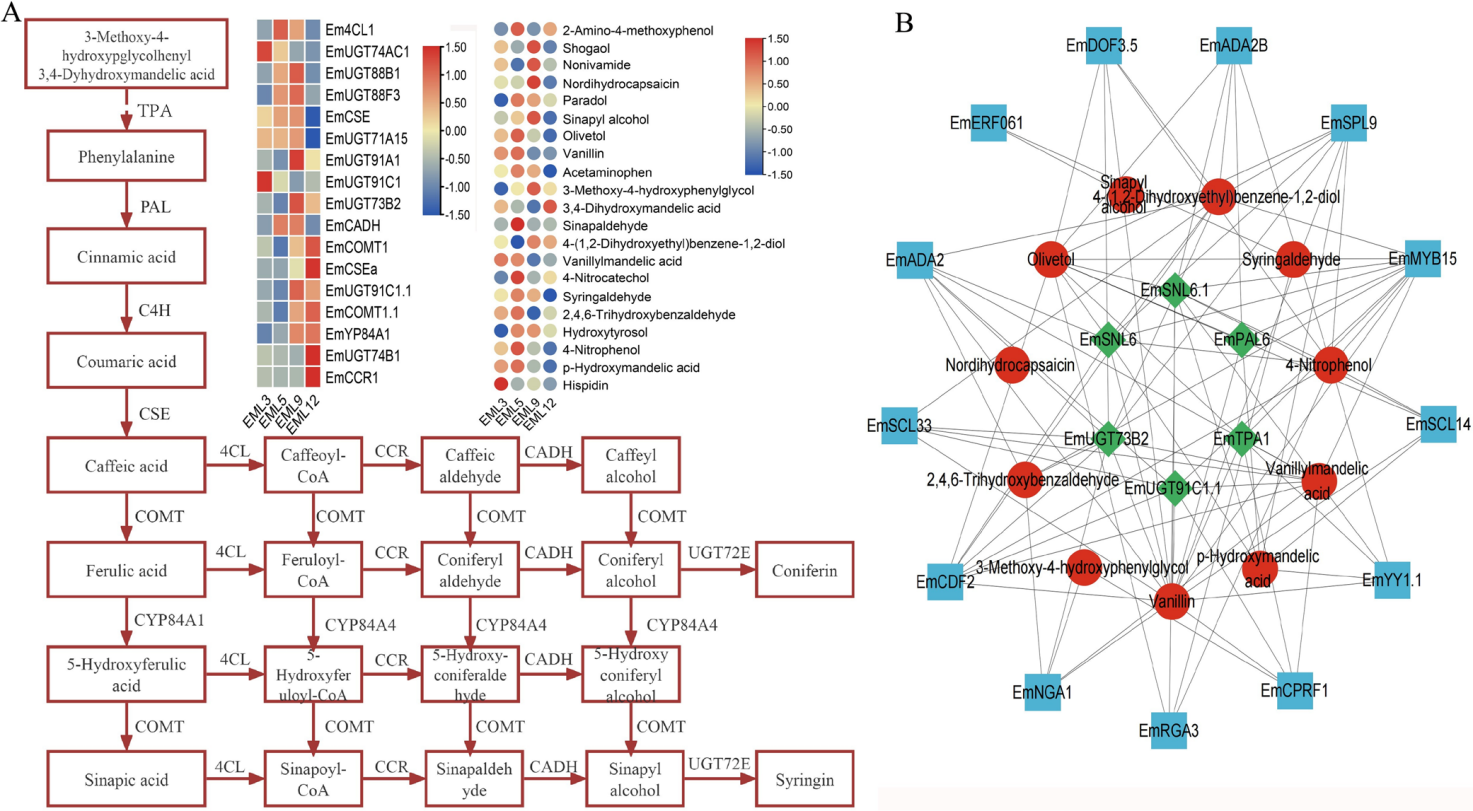
The biosynthesis pathway (A) and the co‐expression network (B) of phenols in EML. Blue square indicated differentially expressed transcription factors, greed rhombus indicated DEGs and red circles indicated DAMs.

**FIGURE 4:**
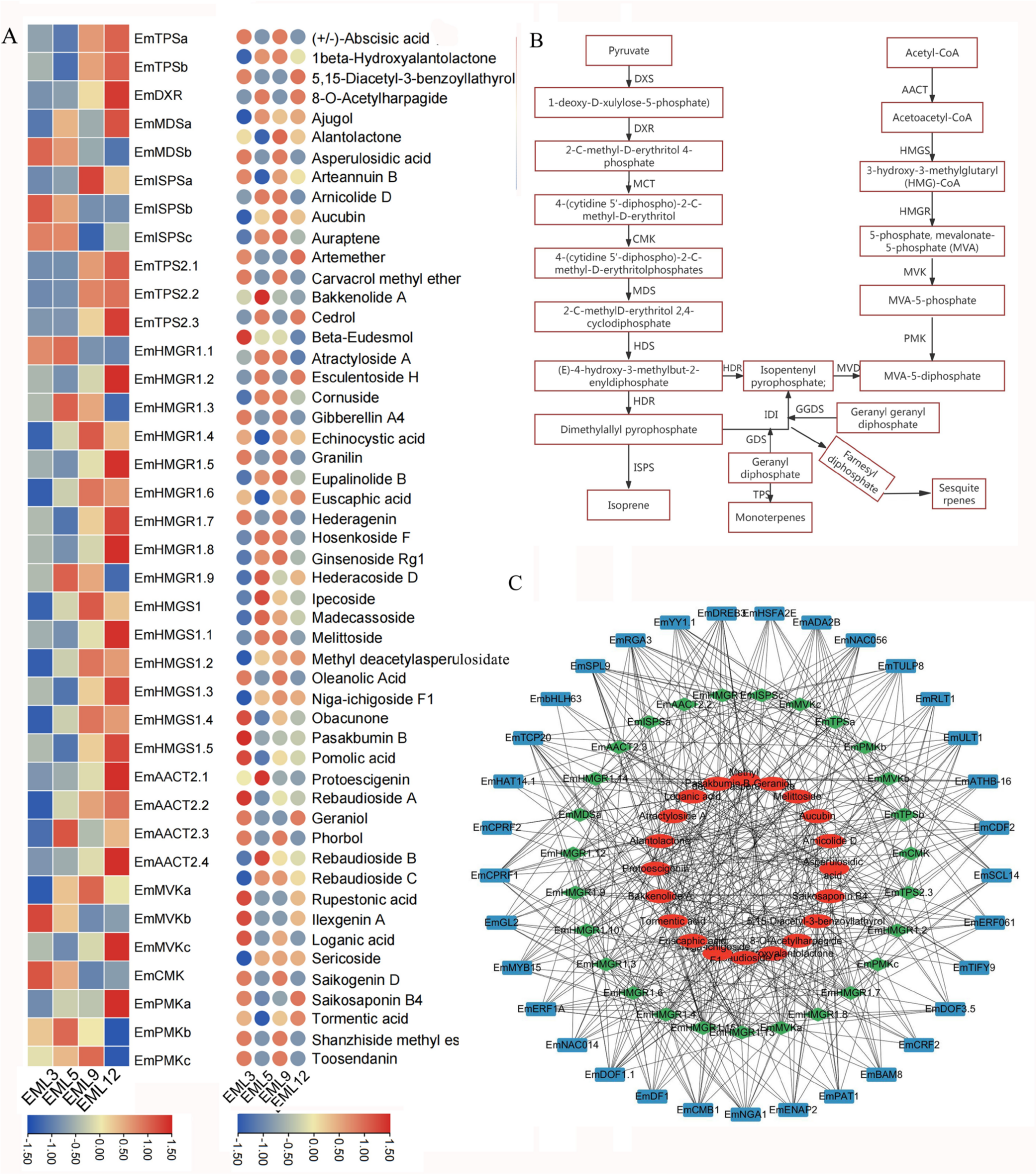
The FPKM value of DEGs and relative content of DAMs (A), the biosynthesis pathway (B) and the co‐expression network (C) of terpenoids in EML. Blue square indicated differentially expressed transcription factors, greed rhombus indicated DEGs and red circles indicated DAMs.

KEGG enrichment analysis of DAMs and DEGs demonstrated that flavonoids biosynthesis‐related pathways were enriched in various EML development stages (Figures S3 and S5). Totally, 32 DAMs were annotated as flavonoids metabolites and enriched in pathways associated with flavonoids biosynthesis, of which rutin, astragalin, quercetin, and luteolin 7‐O‐glucoside were four major flavonoids in EML (Table S3). The 14 DEGs participating in flavonoids biosynthesis pathways included eight families, such as *EmCHSs* and *EmCHIs* (Table S4). For instance, *EmCHS* showed the highest expression level at EML3 and the lowest at EML9, and other genes also illustrated fluctuation expression to play complementary roles in flavonoids biosynthesis (Figure 2A). For phenols and terpenoids, 21 and 52 DAMs, as well as 19 and 37 DEGs were identified to involve in the biosynthesis pathway (Figures 3A and 4A,B). Noticeably, *Em4CL* functioned as a synergistic enzyme both in flavonoids and phenols biosynthesis with relatively high expression level in EML5 and EML9. The relative levels of most DEGs and DAMs illustrated accompany with the content dynamic of flavonoids, phenols, and terpenoids, which directly embodied the contribution of DEGs and DAMs to the accumulation of functional ingredients.

### 2.6 Functional Ingredients Biosynthesis Regulatory Network in EML

To construct the co‐expression network to dissect the regulatory mechanism of functional ingredients biosynthesis, we first employed Pearson's correlation analysis to determine the relatedness among DAMs and DEGs in flavonoids, phenols, and terpenoids biosynthesis pathway with all the tfDEGs. The results revealed significantly positive or negative correlations within a specific pathway (Table S5). For example, *EmPGT1* showed significant correlation with the most number of DAMs, while *EmNAC014* and *EmYY1* with the most DEGs within flavonoids biosynthesis. Similarly, *EmMYB15* and *EmTIFY9* may play certain roles in phenols and terpenoids biosynthesis, respectively. The constructed co‐expression network further illustrated a multi‐regulatory mechanism underlying the biosynthesis of these functional ingredients during EML development (Figures 2B, 3B, 4C).

### 2.7 Verification Analysis of Specific TFs

**FIGURE 5:**
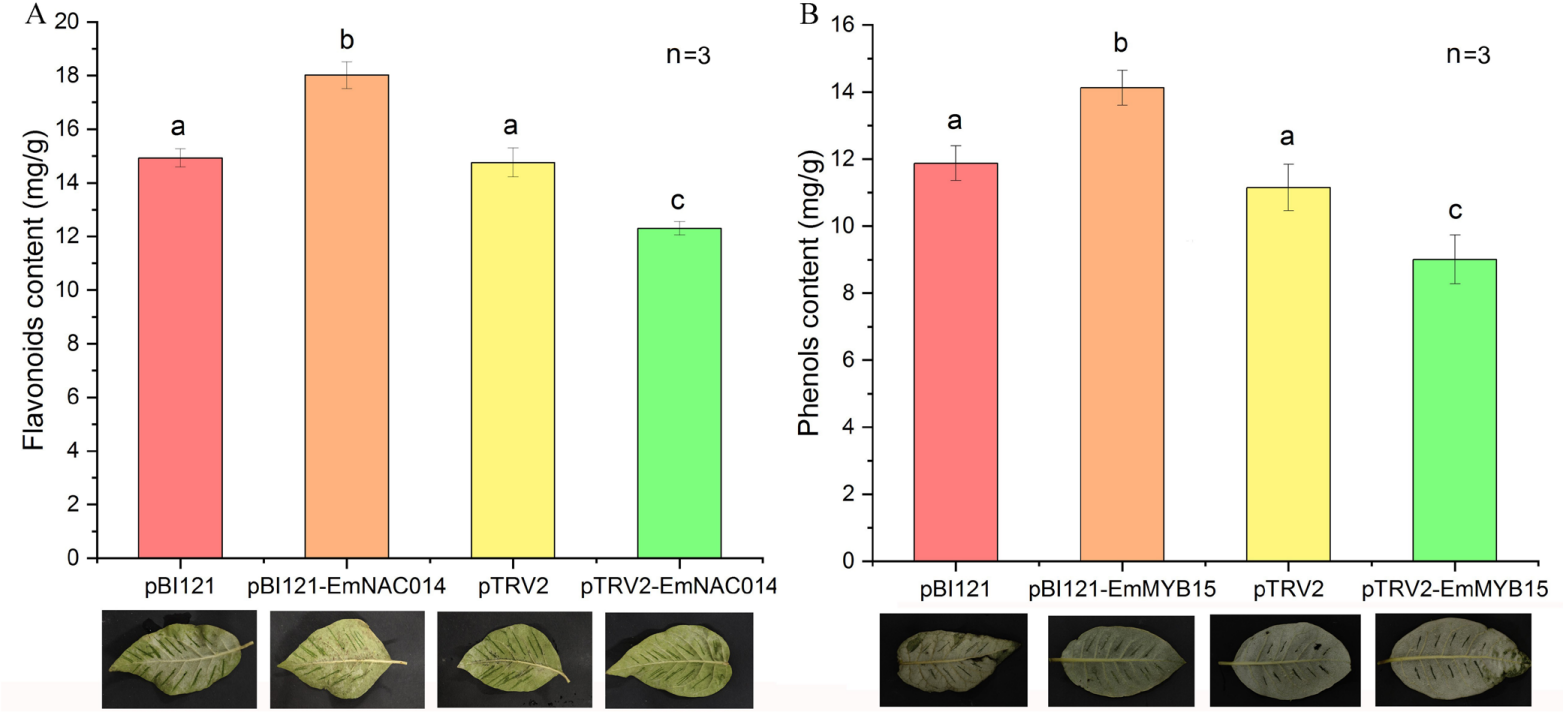
The transient overexpression and VIGS results of *EmNAC014* on flavonoids (A) and *EmMYB15* on phenols (B) contents in EML. Lowercase indicates significant difference.

We successfully produced the overexpression and VIGS plasmid constructs and conducted the transient transformation experiments. Compared with the control, overexpression of *EmNAC014* and *EmMYB15* increased the flavonoids and phenols contents, respectively, and VIGS leaf samples showed the opposite results (Figure 5A,B). This indicated that *EmNAC014* and *EmMYB15* were positive regulators of flavonoids and phenols biosynthesis in EML.

## 3 Discussion

### 3.1 Functional Components in EML and Their Applications

In the present study, we found that the content dynamics of flavonoids, phenols, and terpenoids varied considerably, with the highest content appeared at different developmental stages (Figure 1A,B and Figure S1). We used the normalized relative content of terpenoids to illustrate the changing trends among different EML samples, which resulted from the implication of the metabolome method used. But the quasi‐targeted metabolome used here can detect metabolites of low abundance with higher accuracy (Jiang et al. 2023; Kefale et al. 2023). The flavonoids in EML has attracted more attention with the content varying from 2.314 to 23.42 mg/g (Guo et al. 2011; Li et al. 2015; Liu et al. 2012; Lu, 2014), which was similar with that detected in the present study. Nevertheless, only one or limited EML samples were utilized, and flavonoids content dynamics was not fully explored. Moreover, the exploration of phenols and terpenoids in EML has not been conducted. To our acknowledgment, this paper is the first to demonstrate the accumulation and dynamic pattern of flavonoids, phenols, and terpenoids in EML, hence provides new insights for further utilization of this precious functional trees. The accumulation of flavonoids, phenols, and terpenoids contributed to the antioxidant capacity of EML, which can be attribute to the scavenge and neutralize free radicals, mitigate oxidative stress, and safeguard cells against oxidative damage of these compounds (Romero‐Arias et al. 2019; Zhang, Hou, et al. 2025). The antioxidant capacity of EML can serve as a key determinant of the quality and efficacy, which was reflected by the significant correlation with the functional ingredients contents (Figure 1F). However, DPPH, ABTS and FRAP assays produced divergent antioxidant values for the same sample (Figure 1C–E), which may result from that they probed different chemistry and operated under different conditions, such as reaction mechanisms differentiation, solvent compatibility, steric accessibility, redox potential and metal interference, etc. (Knez et al., 2025; Prior et al. 2005). Therefore, other complementary assays should be combined for a balanced assessment of antioxidant capacity of EML.

Metabolites belong to flavonoids, phenols, and terpenoids, such as epigallocatechin, shogaol and ajugol detected in EML have been reported to exhibit diverse nutritional properties, such as antioxidant and neuroprotective effects (Tao et al. 2024; Wu et al. 2021; Xu et al. 2023). This implies that EML can serve as a functional resource with deep utilization with food and nutrient purposes for various health benefits, such as beverage and additive. For instance, EML beverage processed by blanching and roasting has been common in south Shanxi, which is proven by the masses to be able to lower blood pressure, sugar and lipid, enhance the resistance of capillaries and increase coronary blood flow. Flavonoids, phenols, and terpenoids in EML exhibited favorable biological activities, but their bioavailability is largely affected by structural characteristics, metabolic stability, and absorption efficiency in vivo. Hence, detailed investigation is needed to explore the future utilization of these ingredients.

### 3.2 Accumulation Patterns of Functional Ingredients in EML

EML development involves the differentiated accumulation of various metabolites that influencing the function and nutritional value, which is determined by flavonoids, phenols, and terpenoids metabolites to a great extent. This study revealed that the accumulation of functional ingredients significantly fluctuated, which was in line with the pattern observed in other species. The flavonoids and phenols contents in *Ilex vomitoria* leaf varied among different seasons (Sha et al. 2025). Throughout the development of EML, TFC, TPC, and the terpenoids content all showed relatively high levels at middle to latter development stage. This was consistent with the outside environment change, the high temperature, sunlight and rainfall in summer cause damages to the function of leaf. The accumulation of secondary metabolites can counteract these through reactive oxygen scavenging, decreased lipid peroxidation levels of the cell membrane and protein denaturation (Ren et al. 2025; Zhang, Li, et al. 2025). Noticeably, some of the related DEGs and DAMs showed the highest pattern in late EML development stage close to senescence (Figures 2A, 3A, 4A), which can maintain the stability of internal niche and continuously supply the biosynthesis of these ingredients to cope with the decreasing temperature in late autumn. This may be also the reason for the formation of high tolerance of *E. mollis* to various stresses and be a relict species in the Loss Plateau since Quaternary (Du et al. 2020). Overall, understanding the accumulation and dynamic pattern of functional ingredients in EML will be useful for determining the optimal harvesting time and processing methods to ensure their maximum retention and utilization in related industries (Figure 4).

### 3.3 Flavonoids Biosynthesis and Regulation in EML

The biosynthesis pathway of flavonoids has been investigated in different plant species, and substantial processes have been achieved. Flavonoids biosynthesis starts with the phenylalanine pathway, where phenylalanine ammonia lyase converts phenylalanine to cinnamic acid. CHS is the first critical rate‐limiting enzyme in flavonoids biosynthesis, as it catalyzes the formation of naringenin chalcone (Tohge et al. 2018). Subsequently, under the catalytic action of CHI, naringenin chalcone undergoes cyclization to form naringenin, which is converted to different flavonoids through reactions catalyzed by various enzymes. We found that the structure and properties of flavonoids metabolites in EML were diverse, which resulted from the reticulation of multiple functional genes in the biosynthesis network (Figure 2). The contribution of flavonoids‐related DEGs and DAMs to the content dynamic has been widely revealed in plants. A number of flavonoids biosynthesis genes and metabolites exhibited upregulation during saffron stigma development, which aligned with the observed flavonoids accumulation pattern of this study (Chen et al. 2025). Specifically, numerous TFs, such as MYBs, bZIPs, and NACs, have been identified as important regulators of flavonoids biosynthesis. For instance, *Spatholobus suberectus MYB158* regulated the expressions of *SsC4H*, *SsPAL*, and *Ss4CL* to promote flavonoids biosynthesis and accumulation (Qin et al. 2024). *GbbZIP108* promoted flavonoids content through increasing the transcription levels of several biosynthesis genes in *G. biloba* (Han et al. 2023). In the present study, the co‐expression network analysis revealed that flavonoids biosynthesis was coregulated by several members of TF families, including *EmMYBs*, *EmNACs*, and *EmHSFs*, which showed significant correlation with various flavonoids biosynthesis genes and metabolites (Table S5). *EmHSF24* (evm.TU.Chr02.4713) and *EmNAC014* (evm.TU.Chr01.2151) were significantly correlated with six key genes, including *EmC4H*, *EmF3H*, *EmCHI*, and *EmCHS*, as well as metabolites such as kaempferide and glycitin. HSFs can simultaneously modulate flavonoids biosynthesis and stress tolerance through forming transcription complex (Mishra et al. 2024; Wang et al. 2020; Zhang et al. 2023). Also, NACs were reported to associate with flavonoids biosynthesis in *Ginkgo biloba* (Li et al. 2023), and *PaNAC03* reduced flavonoids content in Norway spruce (Dalman et al. 2017). Recently, Feng et al. (2025) found that *EmMYB05* was the key regulator of flavonoids biosynthesis in *E. mollis* kernel. We proved the positive regulatory role of *EmNAC014* in EML flavonoids accumulation with transient overexpression and VIGS analysis, which illustrated the tissue‐specific regulatory manner of flavonoids biosynthesis. Therefore, further investigation is required to determine the interaction and downstream effects of *EmNAC014* to explicitly illustrate the biosynthesis mechanism of flavonoids in EML.

### 3.4 Biosynthesis and Regulation of Phenols and Terpenoids in EML

Phenols and terpenoids have been widely recognized as crucial bioactive components in plants (Ding et al. 2025; Jin et al. 2025). The phenols and terpenoids biosynthesis pathway is intricate and consists of various sequential enzymatic reactions that have been comprehensively investigated (Ali et al. 2018; Chu et al. 2020). PAL is a rate‐limiting enzyme in the biosynthesis of phenols and we identified seven *EmPALs* in the transcriptome data, and four were defined as DEGs (evm.TU.Chr01.6097, evm.TU.Chr04.448, evm.TU.Chr09.486 and evm.TU.Chr13.2555). All the *EmPALs* illustrated fluctuated expression level, only evm.TU.Chr01.6097 showed the highest expression level at EML9 and EML12 with approximately 10 times of other *EmPALs*. This indicated that evm.TU.Chr01.6097 may be the key member in *EmPALs*, which was consistent with the general phenomenon in plants that some key genes played major roles in a specific family with higher expression than other paralogs (Du et al. 2025; Zhao et al. 2023). From EML1 to EML4, TPC gradually increased and stably fluctuated during EML5 to EML12, this pattern showed a substantial linear association with the FPKM value of evm.TU.Chr01.6097. The rise in gene expression and the corresponding enzymatic activities of *EmPALs* and the gradual accumulation of metabolites, such as digallic acid and 3,4‐dihydroxy phenylethanol, accounted for the increase of TPC in EML to a great extent, as was unvealed in Chinese wild rice and barley seedlings (Chu et al. 2019; Tian et al. 2024). Besides *EmPALs*, some phenols biosynthesis genes showed significant correlation with corresponding metabolites, including one *Em4CL*, one *EmCCR*, two *EmCOMT*, and six *EmUGTs* (Table S4). In the biosynthesis of phenols, 4CL catalyzes the ligation of CoA to various phenolic acids, which are subsequently converted to hydroxycinnamoyl‐CoA esters and then to hydroxycinnamaldehydes under the catalysis of EmCCR. CCRs mainly function in monolignol biosynthesis, and numerous studies revealed that CCRs can regulate lignin biosynthesis and the resistance of plants to various diseases and insect infection (Liu et al. 2025; Rao et al. 2022). The expression of *EmCCR* during EML development may elucidate the high resistance of *E. mollis* to various external stresses to some extent. Furthermore, the involvement and the high expression of *EmCCR* in phenols biosynthesis is general in plants (Li, Wu, et al. 2025), which mirrored the simultaneous multi‐functions in metabolic pathways. Within all the TFs correlated with phenols biosynthesis genes in EML, we found that *EmMYB15* significantly associated with the most genes, including *EmSNL6* (evm.TU.Chr05.2226), *EmTPA1* (evm.TU.Chr08.205), and *EmUGT91A1* (evm.TU.Chr03.4412) (Table S5). It was found that R2R3‐MYB subgroup 7 factors directly targeted to the promoter of *AtUGT91A1* to regulate secondary metabolites biosynthesis (Stracke et al. 2007). The transient overexpression and VIGS results showed that *EmMYB15* can positively regulate phenols biosynthesis in EML, which meant that *EmMYB15* may bind to the promoter of these three function genes to regulate their expression and the biosynthesis of phenols. This needed further verification and suggested the evolutionary conservation of some TFs during the diversification of Elaeagnaceae and Brassicaceae. Moreover, within all the phenols metabolites, arbutin is a phenolic glucoside widely revealed in different plant species, and has valuable health benefits for human beings, such as protecting liver from alcohol‐induced injury (Benkovic et al. 2021; Radmard et al. 2021). Furthermore, hydroxytyrosol acetate is a phenolic compound widely found in another typical oil crops *Olea europaea* , which exhibits oxidation resistance, heart protection, and antibacterial activity (Wei et al. 2018, 2024). Owing to its health benefits, hydroxytyrosol acetate has been widely applied as functional food additions and nutraceuticals. Therefore, EML may serve as a new source of natural phenols resulting from the rich content of arbutin and hydroxytyrosol acetate.

The MVA and MEP are two major precursor metabolic pathways for terpenoids biosynthesis (Jin et al. 2025; Nagegowda 2010). Also, the MEP pathway provides phytyl‐diphosphate or geranylgeranyl diphosphate for vitamin E biosynthesis in *E. mollis* (Du et al. 2024), which indicated that vitamin E biosynthesis may also be active to some extent in EML. Totally, we detected 103 functional genes associated with terpenoids biosynthesis in EML, of which 25 were defined as DEGs (Table S4). The fluctuated expression of these genes led to the increased diversity of the terpenoids during EML development. Noticeably, TPSs have been widely evidenced to play certain roles in terpenoids biosynthesis in various plant species. For example, *AtTPS21* directly regulated the formation of (E)‐β‐stigmastigmine to influence the biosynthesis of sesquiterpenes in *A. thaliana* (Isemer et al. 2012). *CmTPS03*, *CmTPS21*, and *CmTPS* were found to be key genes in regulating terpenoids biosynthesis in tea used chrysanthemum cultivars (Yu et al. 2025). Hence, *EmTPSs* may serve as potential key candidates in EML terpenoids biosynthesis. Furthermore, as an important terpenoids biosynthesis gene, it has been recognized that various TFs can directly bind to the *cis‐elements* of HMGRs to regulate their expression, ultimately influencing terpenoids biosynthesis (Devi et al. 2024; Ma et al. 2024; Wei et al. 2023). For instance, *WsAP2* crucially regulated the expression of genes in the terpenoids pathway via induction of expression of *HMGRs* in *Withania somnifera* (Tripathi et al. 2020). Additionally, *EmTIFY9* showed a significantly negative correlation with *EmTPS*, which was similar to the situation observed in *Aquilaria sinensis* . Liao et al. (2023) reported that overexpression of *AsJAZ1*, a tify domain‐containing gene, significantly down‐regulated the expression of sesquiterpene synthase genes *TPS21* and *TPS11*, suggesting that TIFYs might serve as depressors in terpenoids biosynthesis in EML. Moreover, among the identified TFs involved in terpenoids biosynthesis, three TFs, including *EmDOF1.1*, *EmDREB3*, and *EmSPL9*, showed relatively high significance (Table S5). The progressive regulation of the miR156/SPLs complex in terpenoids biosynthesis has been widely uncovered (Yu et al. 2015; Zhang, Ni, et al. 2025), the involvement of *EmSPL9* in EML terpenoids biosynthesis may be a conserved evolutionary strategy of SPLs in Elaeagnaceae. The uniquely regulatory role of DREBs in terpenoids biosynthesis was universally identified; for instance, LITCHI017494, a DREB family in *Litchi chinensis* , directly regulated terpenoids biosynthesis by activating tandemly repeated *LcTPSs* (Wu et al. 2025). The above‐revealed regulators contributing to terpenoids biosynthesis in EML may need further testimony in combination with numerous methods, which can provide valuable biomarkers for further genetic breeding of these important newly developed function trees.

## 4 Conclusion

In the present study, we quantified the flavonoids and phenols of EML in various development stages. The significant correlation between antioxidant capacities and ingredients contents contributed to the quality of EML, indicating that EML can serve as a novel resource with food and nutritional purpose. Furthermore, we identified functional genes, metabolites and regulators involved in flavonoids, phenols, and terpenoids biosynthesis in EML, thereby enhancing our understanding of functional ingredients biosynthesis mechanism in *E. mollis* . The co‐expression network analysis unraveled the presence of MYBs, NACs and AP2/ERFs that potentially function to play regulatory roles. The specific roles of *EmNAC014* and *EmMYB15* in flavonoids and phenols biosynthesis were preliminarily testified. Further studies should be conducted to validate the underlying regulatory role of these TFs. Overall, the comprehensive analysis of accumulation dynamics, biosynthetic pathways and regulatory mechanisms of flavonoids, phenols, and terpenoids will be vital for enhancing their yield and quality in *E. mollis* , but also offers valuable insights for the development of innovative functional foods.

## Author Contributions

**Song Jia:** investigation, funding acquisition, validation. **Yu Wendong:** investigation. **Wu Jing:** investigation, resources. **Su Yanping:** writing – original draft, formal analysis. **Wu Zhenzhen:** investigation. **Huang Yuyin:** investigation. **Hu Xiaoyan:** investigation, software, formal analysis, writing – original draft. **Qiu Qiandong:** investigation. **Du Shuhui:** writing – review and editing, conceptualization, supervision.

## Funding

This research was supported by Fundamental Research Project in Shanxi Province (202503021211161), Special Project for Science and Technology Cooperation and Exchange of Shanxi (202404041101034), and Key R&D Project in Hebei Province (20326335D).

## Conflicts of Interest

The authors declare no conflicts of interest.

## Supporting information


**Figure S1:** The relative content of terpenoids during EML development.
**Figure S2:** The correlation of quality charge samples (A), PCA (B) of metabolome data and the annotated metabolites in EML (C).
**Figure S3:** The KEGG enrichment analysis of DAMs in the three comparisons of EML.
**Figure S4:** The PCA (A), correlation analysis of transcriptome data (B) and qRT‐PCR results.
**Figure S5:** The KEGG enrichment analysis of DEGs in the three comparisons of EML.

**Table S1:** The primers used in the present study.
**Table S2:** The FPKM value of differentially expressed transcript factors identified in transcriptome data.
**Table S3:** The identified DAMs related to flavonoids, phenols, and terpenoids in metabolome data.
**Table S4:** The DEGs involved in flavonoids, phenols, and terpenoids biosynthesis.
**Table S5:** The filtered correlation results among differentially expressed TFs, DEGs and DAMs.

## Data Availability

The clean data of transcriptome analysis were deposited in the Genome Sequence Archive in National Genomics Data Center, China National Center for Bioinformation/Beijing Institute of Genomics, Chinese Academy of Sciences (GSA: CRA027007) that are publicly accessible at https://ngdc.cncb.ac.cn/gsa. The self‐assembled *E. mollis* genome was deposited in NCBI with accession number PRJNA1272808.
