# Supplementary material for: Integrated Analysis Reveals Functional Ingredients Biosynthesis and Regulatory Mechanisms in Elaeagnus mollis Diels Leaf
Source: Food Sci Nutr. 2026 Mar 25;14(3):e71679. doi: 10.1002/fsn3.71679 (PMC13093282; doi:10.1002/fsn3.71679)
Supplement: Supplementary file 1 — Figure S1: The relative content of terpenoids during EML development. Figure S2: The correlation of quality charge samples (A), PCA (B) of metabolome data and the annotated metabolites in EML (C). Figure S3: The KEGG enrichment analysis of DAMs in the three comparisons of EML. Figure S4: The PCA (A), correlation analysis of transcriptome data (B) and qRT‐PCR results. Figure S5: The KEGG enrichment analysis of DEGs in the three comparisons of EML. [file FSN3-14-e71679-s002.docx]

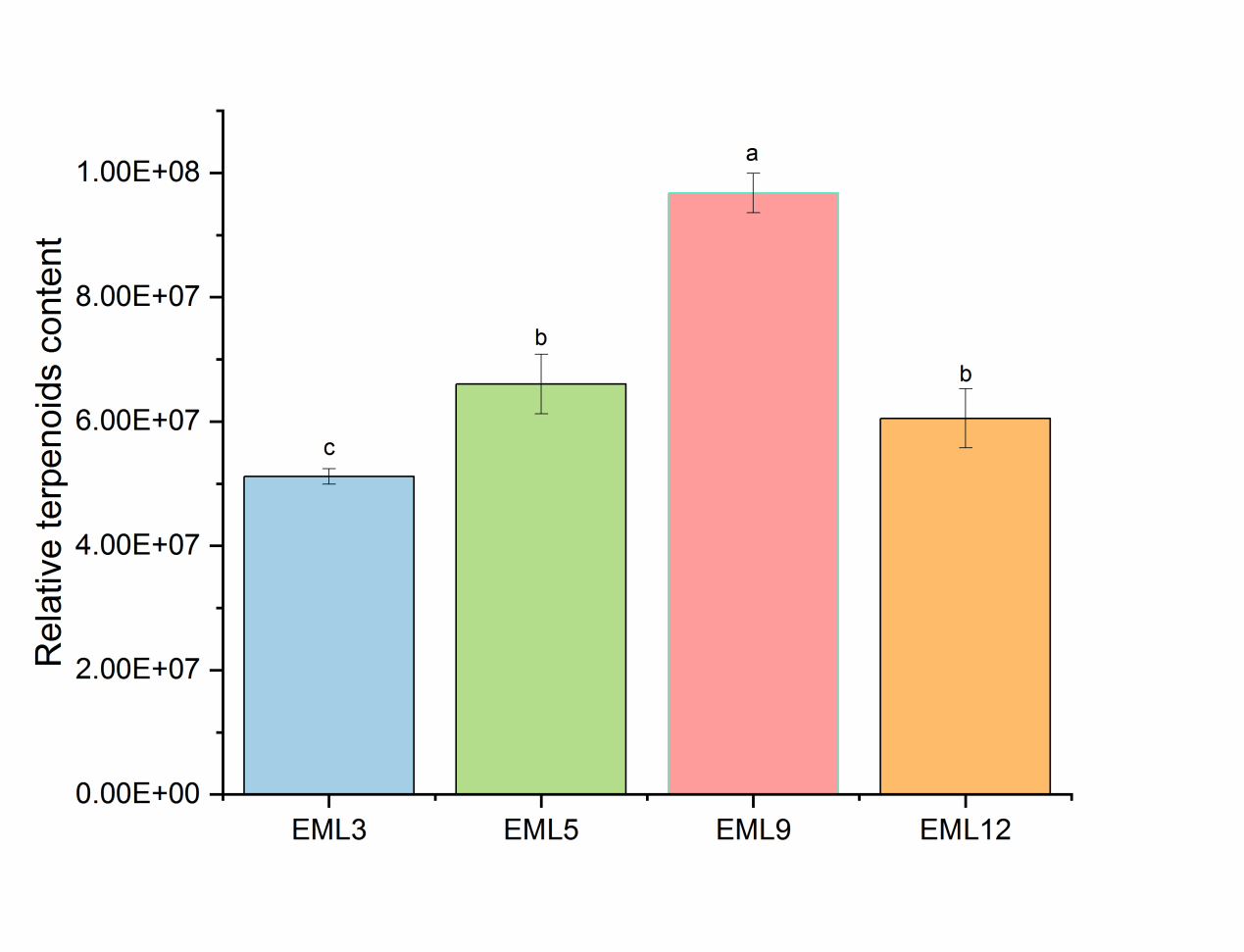


n=3

Figure S1 The relative content of terpenoids during EML development

Note: lowercase indicated significant difference.


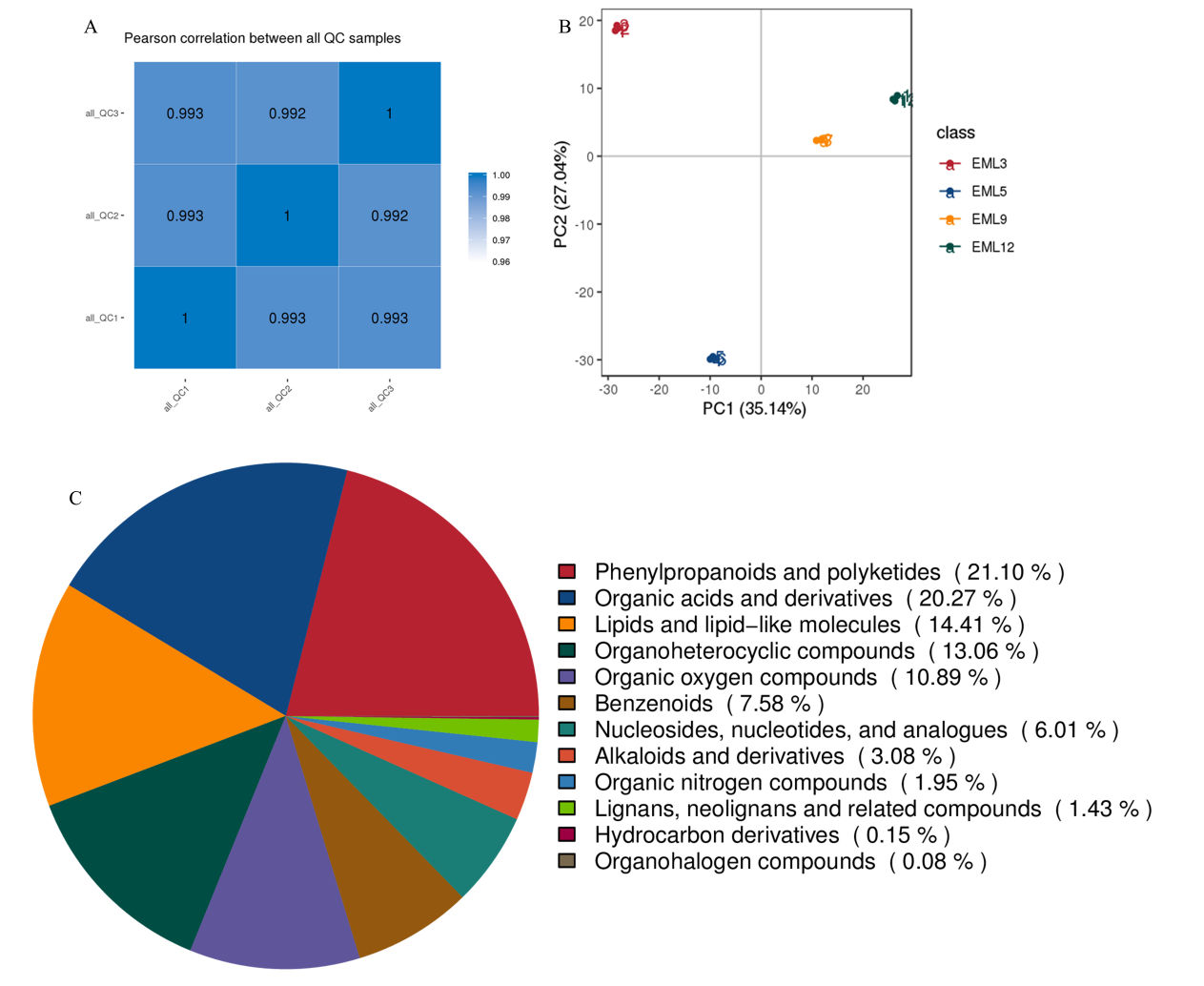


Figure S2 The correlation of quality charge samples (A), PCA (B) of metabolome data and the annotated metabolites in EML (C).


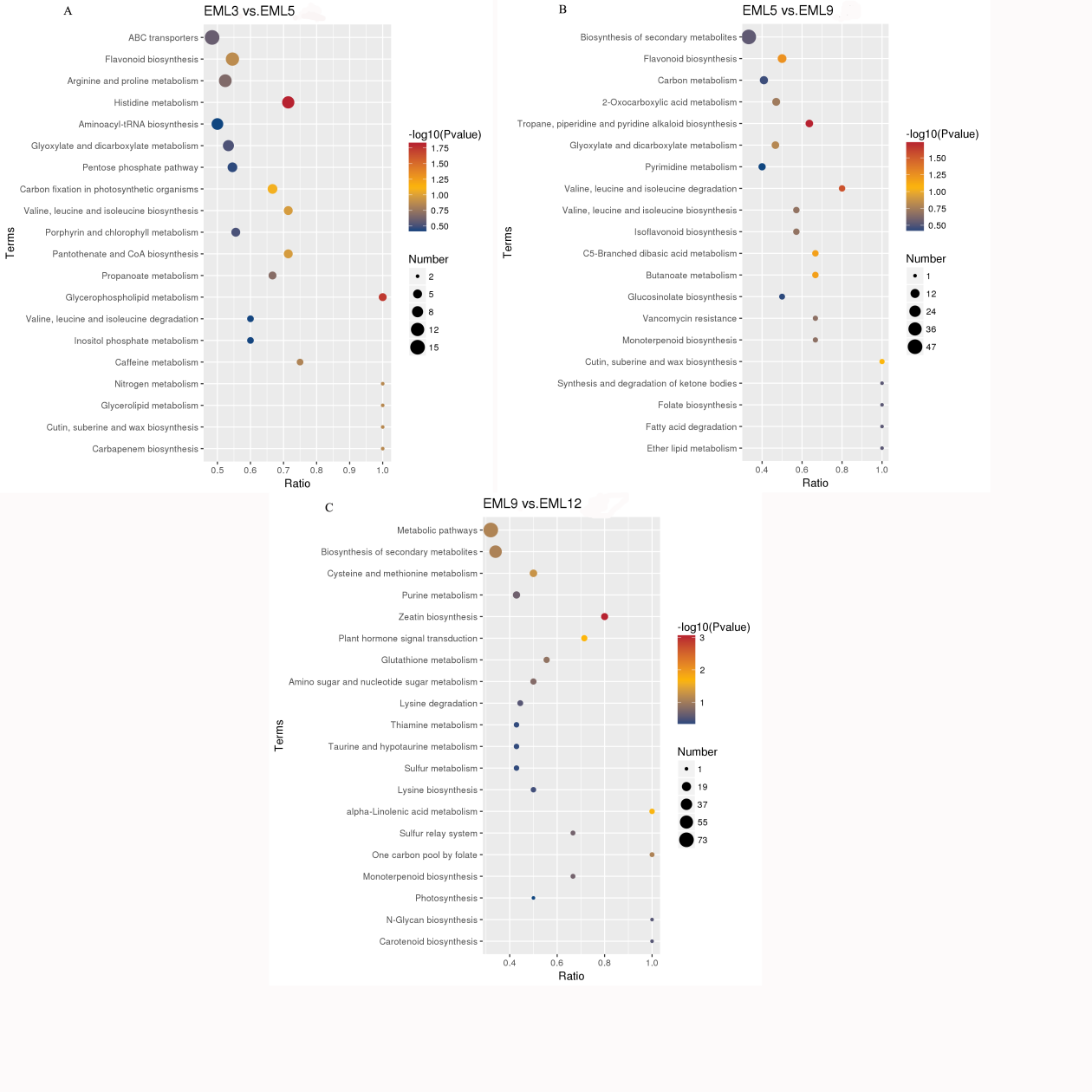


Figure S3 The KEGG enrichment analysis of DAMs in the three comparisons of EML


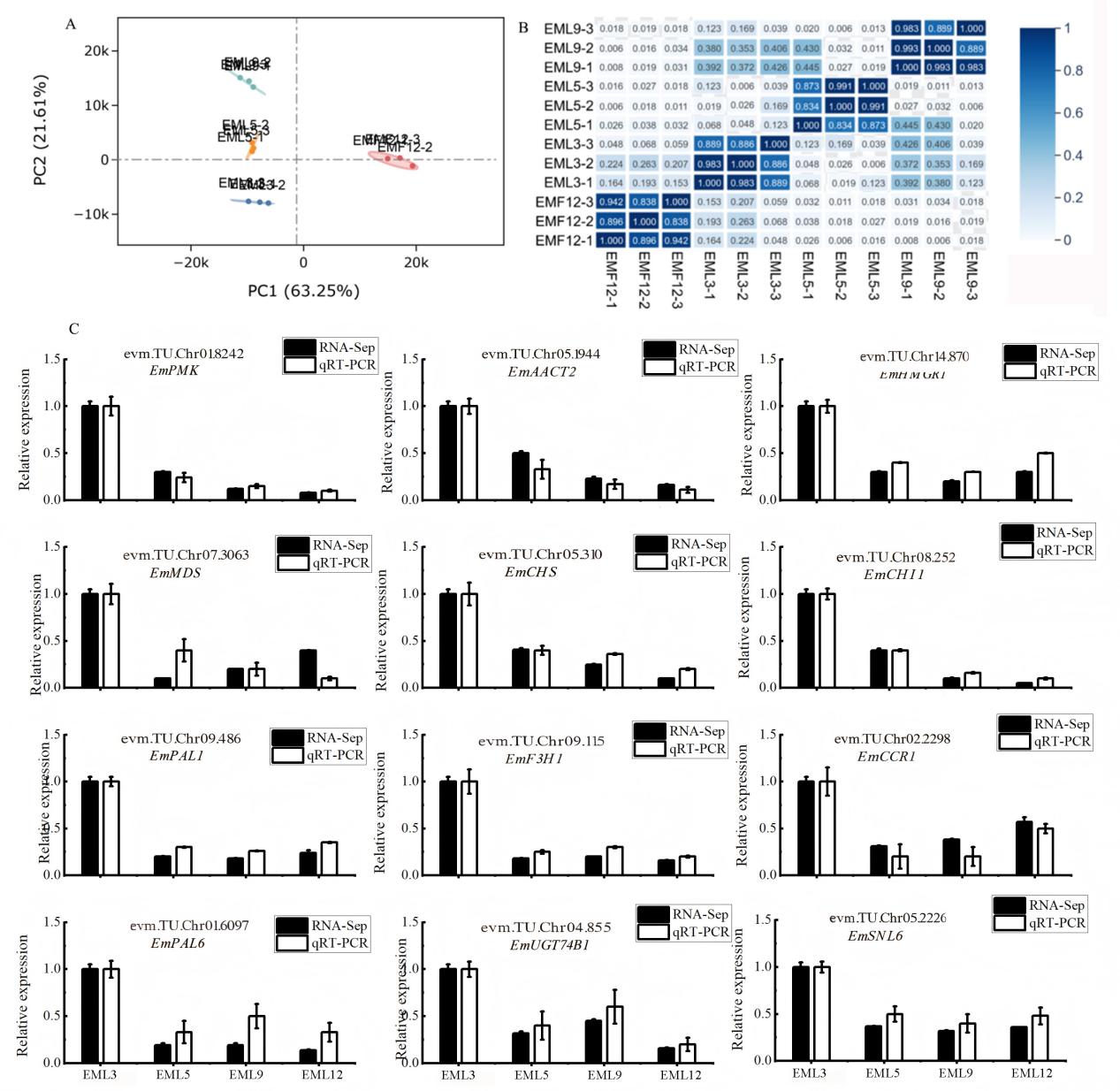


Figure S4 The PCA (A), correlation analysis of transcriptome data (B) and qRT-PCR results.


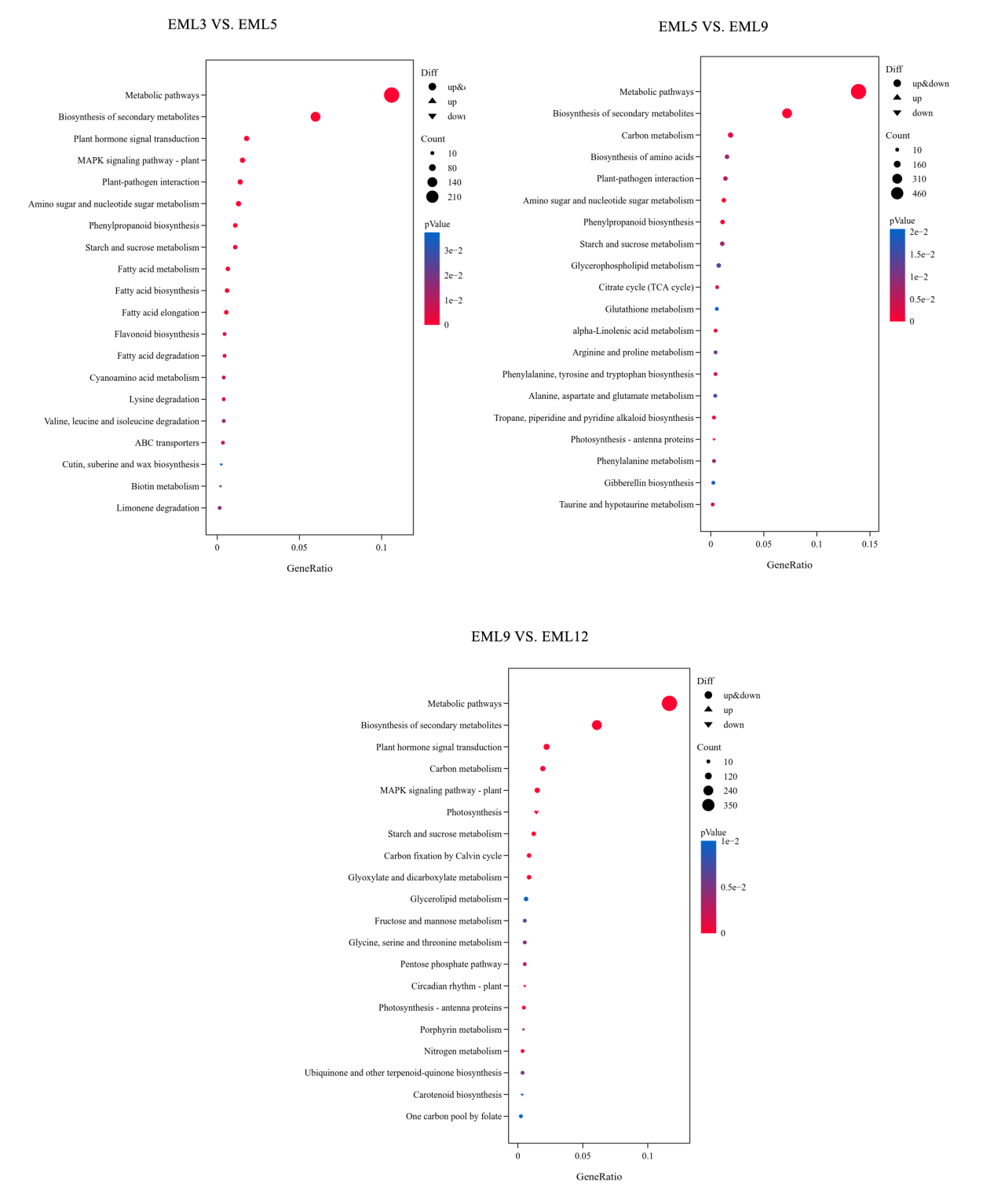


Figure S5 The KEGG enrichment analysis of DEGs in the three comparisons of EML
